# Membrane nanodomains and transport functions in plant

**DOI:** 10.1093/plphys/kiab312

**Published:** 2021-07-13

**Authors:** Alexandre Martinière, Enric Zelazny

**Affiliations:** BPMP, Univ Montpellier, CNRS, INRAE, Institut Agro, Montpellier, France

## Abstract

Far from a homogeneous environment, biological membranes are highly structured with lipids and proteins segregating in domains of different sizes and dwell times. In addition, membranes are highly dynamics especially in response to environmental stimuli. Understanding the impact of the nanoscale organization of membranes on cellular functions is an outstanding question. Plant channels and transporters are tightly regulated to ensure proper cell nutrition and signaling. Increasing evidence indicates that channel and transporter nano-organization within membranes plays an important role in these regulation mechanisms. Here, we review recent advances in the field of ion, water, but also hormone transport in plants, focusing on protein organization within plasma membrane nanodomains and its cellular and physiological impacts.

## Teaser

This article reviews our current knowledge about the role of membrane organization and dynamics for transport functions in plants.

## Introduction

The plasma membrane (PM) is composed of lipids and proteins and forms a selective barrier between cell interior and the extracellular environment. The semi-permeability of the PM permits a tight control of solute and macromolecules passage through the action of specific transporters and channels that are highly regulated to maintain cell homeostasis and ensure proper plant growth and development.

**Advances**

Since the fluid mosaic model of membranes proposed in the 1970s, our view of biological membranes became more and more complex (Singer and Nicolson, 1972; Nicolson, 2014). It is now admitted that plant membranes are composed of a juxtaposition of domains of different types and sizes that differ in their protein and lipid compositions (Gronnier et al., 2018; Jaillais and Ott, 2020). This vision of membrane organization is illustrated by the work of Jarsch and co-workers showing the coexistence of a plethora of highly distinct subdomains in the PM of plant cells (Jarsch et al., 2014). Those structures can be classified depending on their size. Thus, nanodomains are defined as submicron protein and/or lipid assemblies (20 nm to 1 µm), whereas microdomains correspond to larger assemblies with a size above 1 µm as exemplified by cell polar domains, the Casparian strip domain or plasmodesmata (Ott, 2017; Jaillais and Ott, 2020). The concept of membrane partitioning was raised at first using biochemical approaches. Indeed, due to the highly ordered structure of certain nanodomains, specific membrane fractions called detergent-resistant membranes (DRMs) can be isolated. They are not solubilized by nonionic detergents such as Triton X100 in the cold and show an enrichment in sterols, sphingolipids, and associated proteins (Mongrand et al., 2004; Laloi et al., 2007; Lefebvre et al., 2007). Therefore, DRM constitute a biochemical way to investigate the association between specific lipids and proteins within the PM. Note that this approach was particularly efficient to identify a set of nanodomain-resident proteins, which will be detailed later in this review. Interestingly, the protein content of plant DRM was shown to be modified by biological stimuli (Keinath et al., 2010). In the past, however, DRM preparation was proposed by some to not reflect the in vivo repartition of lipids and proteins (Shogomori and Brown, 2003; Tanner et al., 2011). In addition, the binary nature of DRM fractionation (either in or out of DRM) leads to an oversimplification of membrane heterogeneity and consequently DRM only represent a subpart of the diversity of lipids and proteins present in membrane nanodomains. Although some caution should be undertaken when analyzing DRM lipidomic and proteomic data, they constitute an interesting starting point to investigate plant nanodomain functions. In addition, live-cell imaging microscopy appears to be an essential complementary approach to study nanodomains, especially regarding their dynamics. Until recently, due to light diffraction limit, objects of small size (around ∼250 nm) were not resolvable by microscopy, which restricted the observation of nanometric structures. The so-called super-resolution microscopy techniques overcame this issue and are now classically used in animal biology and emerge in plant field, promoting the description of membrane organization (Komis et al., 2015).

- The concept of nanodomain organization of proteins could be expanded to include transport functions in plants.
- Nanodomains regulate internalization of PM transporters in response to cell stimuli.
- Nanodomains allow co-clustering of proteins with their regulators.

In this review, after technical considerations on advanced microscopy approaches recently applied to plant samples, we will illustrate the role of nano-organization of membranes in the regulation of molecule transport in plants. We will mainly focus our attention on PM nanodomains resulting from the functional assembly of sterols, sphingolipids, and specific proteins although it is important to mention that the lipid composition of nanodomains appears to be multiple. This was recently illustrated in a work showing that an anionic lipid, e.g. phosphatidyl-serine, mediates Rho GTPase signaling through nanometric structures in the PM (Platre et al., 2019).

## Technical advances in plant microscopy to study membrane nanodomain organization

**Figure 1:**
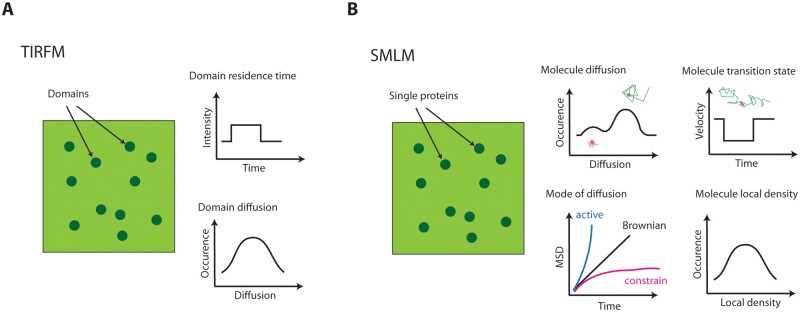
Comparison of the type of data obtained with total internal reflexion fluorescence microscopy (TIRFM) and SMLM techniques. A, Under TIRFM, the number of fluorescent proteins in a given fluorescence-emitting domain cannot be determine experimentally. The duration of the signal can be related to the domain on–off at the PM (graph titled domain residence time). Domain displacement can also be documented (graph titled domain diffusion) and informs about the domain spatial dynamics. B, With SMLM, each single fluorescent dot corresponds to an individual emitter. Its individual diffusion can be estimated and may reflect some heterogeneity since molecules with high diffusion can co-exist with molecules of lower diffusion (graph titled molecule diffusion). MSD plot informs about the diffusion mode that could be either normal (Brownian) or abnormal (constrained or active; graph titled mode of diffusion). SMLM can also be used to determine molecule transition state. Typically, plot of velocity would inform about the molecule displacement for each time point of the track and reveals change of diffusion behavior along time (graph titled molecule transition state). This is particularly valuable to study protein recruitment in membrane nanodomains. To some extent, SMLM can also be used to study protein spatial organization. Localization of molecules along time can serve to calculate their local density (graph titled molecule local density), which can reveal where proteins form clusters and can be compared to TIRF observations

Point scanning confocal microscopy presents a limited resolution in z and, consequently, classically displays a relatively low signal-to-noise ratio when observing plant PM. Historically introduced in the field of plant biology by the Bednarek laboratory, total internal reflection fluorescence (TIRF) microscopy is based on the use of a laser illumination that goes under total reflection at the interface of two media of different refraction indexes (Konopka et al., 2008; Konopka and Bednarek, 2008). This creates an electromagnetic field, called evanescent wave, which is spatially concentrated in the vicinity of the illumination source and propagates parallel to the plane of the interface. It results in a smaller illumination in z compared to confocal and a higher time resolution (Axelrod et al., 1983). TIRF allows the visualization of fluorescently tagged proteins only when they are in or in very close proximity to the PM and constitutes a technique of choice to study protein dynamics, making accessible quantitative parameters like the residence time in membranes but also the displacement of protein clusters (Figure 1A, top and bottom panels on the right). In some cases, variable angle epifluorescent microscopy (VAEM) rather than true TIRF was used on plant samples. In this case, the illumination beam is set below the critical angle to obtain an oblique illumination of the cell. This leads to a better signal-to-noise ratio compared to epifluorescence and allows getting signal a bit deeper in cells and consequently could overcome cell wall thickness. TIRF or VAEM was historically used to analyze clathrin-mediated endocytosis (CME) but were also very useful to investigate the organization and the dynamics of membrane nanodomain-localized proteins such as Plasma Membrane Intrinsic Protein 2;1 (PIP2;1), PIN-Formed 2 (PIN2), Brassinosteroid Insensitive 1 (BRI1), Flagellin Sensitive 2 (FLS2), Respiratory Burst Oxidase Homologue D (RbohD), Ammonium Transporter1 (AMT1), and Nitrate Transporter 1.1 (NRT1.1; Kleine-Vehn et al., 2011; Li et al., 2011; Wang et al., 2013; Hao et al., 2014; Martins et al., 2015; Bücherl et al., 2017; Zhang et al., 2019; Narasimhan et al., 2020). All these proteins were described to cluster in membrane nanodomains of a size close to the optical resolution (300 nm).

Super-resolution microscopy shows increased resolution compared to other light microscopy technics. Over the years, numerous approaches were developed to circumvent light diffraction. They can be divided into two main groups. The first one exploits the nonlinearity of emitters’ response to excitation. This is including techniques based on interference pattern (structure illumination microscopy), point scanning or spinning-based super resolution (airyscan; super resolution by re-assignment), fluctuation analysis methods (super-resolution radial fluctuations), and also stimulated emission depletion microscopy, where emission beam is overimposed by a donut-shaped depletion laser (Hell and Wichmann, 1994; Baddeley et al., 2007; Sheppard et al., 2013; Huff, 2015; Culley et al., 2018). The second group of technic, usually called single molecule localization microscopy (SMLM), is based on stochastic emission. This is allowing a separation in time of each emitter to prevent Airy disk overlap. To achieve it, emission often is manipulated by photoconversion, photoswitching, bleaching, or blinking (Betzig et al., 2006; Hess et al., 2006; Rust et al., 2006). The localization of a high number of proteins is reachable by iterative activation/bleaching cycles and the recording of each molecule on one by one base (Betzig et al., 2006; Hess et al., 2006). Since some of the SMLM techniques, such as photo-activated localization microscopy are compatible with live imaging, the mobility of single molecules can be tracked in the cell before their fluorescence bleaching. Consequently, quantitative information about protein diffusion and localization is obtained (Manley et al., 2008). With this technic, called single particle tracking photoactivated localization microscopy (sptPALM), the instantaneous diffusion coefficient of each molecule can be estimated (Figure 1B, molecule diffusion) and it is also possible to determine its type of diffusion that can be either Brownian, constrained (e.g. when a protein is retained in a domain), or directed when molecule diffusion is enhanced by flow (e.g. transport along cytoskeleton) by the calculation of molecule mean square displacement (MSD; Figure 1B, mode of diffusion). MSD is a common measurement of deviation of the position of a particle over time. It is used to distinguish between a random movement, the presence of advective forces or constrains to diffusion. Interestingly, SMLM can also be used to study transition between those different diffusion behaviors, like for diffusible molecules that are transiently trapped in membrane nanodomains (Figure 1B, molecule transition state). From SMLM data, it is also possible to study protein or lipid clustering. Therefore, local molecular density can be estimated as proposed, for example, by Levet et al. (2015; Figure 1B, molecule local density). These data are really valuable to understand the membrane dynamic partitioning and its impact on protein function (Nicovich et al., 2017). Super-resolution microscopy has revolutionized biological imaging during the past decades and is increasingly used in the plant biology field as illustrated by the imaging of proteins localized in plasmodesmata (Fitzgibbon et al., 2010), in the PM (Kleine-Vehn et al., 2011; Retzer et al., 2017) and in the trans-Golgi network (Uemura et al., 2014), cell wall components (Liesche et al., 2013; Haas et al., 2020), and microtubules (Dong et al., 2015). Note that the recent development of sptPALM to plant samples was particularly powerful to study membrane organization (Hosy et al., 2015; Bayle et al., 2021). Since sptPALM can be used on living cells, it allows the analysis of the dynamics and the heterogeneous lateral segregation of individual proteins and lipids in the PM (Hosy et al., 2015; Gronnier et al., 2017; Perraki et al., 2018; Martinière et al., 2019; Platre et al., 2019; Smokvarska et al., 2020). Therefore, sptPALM is probably one of the most valuable tools to dissect membrane structures and their influence on protein function. It is important to mention that SMLM approaches and TIRF microscopy can be complementary as exemplified by the study of the intracellular dynamics of Clathrin Light Chain 2 (CLC2) in plants. Under TIRF microscopy, fluorescently tagged CLC2 appeared as laterally stable punctuated structures in the PM and the residence time of CLC2 foci was used to estimate the duration of clathrin vesicle formation and release from the PM (Konopka et al., 2008; Gadeyne et al., 2014; Dejonghe et al., 2016). Although by SMLM, the vast majority of CLC2 molecules have a low diffusion and might correspond to the CLC2 foci observed by TIRF, other CLC2 molecules in the same cell show a much higher diffusion (Martinière et al., 2019). This last population of molecules is not observable under TIRF microscopy and could correspond to CLC2 proteins in association with adaptor proteins diffusing in the membrane before the formation of clathrin vesicles. These data illustrate how the dynamics of nanodomains, e.g. CLC2 foci, and single molecules, e.g. individual CLC2, can differ (Figure 1).

## Nanodomain-resident proteins

Some proteins are exclusively associated with DRM and only localize in membrane nanodomain structures, as revealed by microscopy techniques. In plants, these nanodomain-resident proteins include the Remorins (REMs), that are specific to plants, and the superfamily of Stomatin/Prohibitin/Flotillin/HflK/C (SPFH) domain-containing proteins. Some REMs and SPFH proteins have been widely used as nanodomain markers in imaging and biochemical approaches in plants. Here, we will give a brief overview of some important features of REM and SPFH proteins, for more details, readers can refer to recent reviews on this field (Daněk et al., 2016; Gronnier et al., 2018; Gouguet et al., 2020). So far, REMs were the best-characterized nanodomain-resident proteins in plants. At the subcellular level, REM were notably shown to be targeted to the cytosolic leaflet of the PM through a C-terminal lipid-binding motif called the C-terminal Anchor (CA; Konrad et al., 2014; Gronnier et al., 2017; Perraki et al., 2018). Interestingly, both sterol and phosphatidylinositol 4-phosphate are required for the nanodomain organization of potato (*Solanum tuberosum*) StREM1;3 in the PM (Gronnier et al., 2017). Importantly, some Arabidopsis (*Arabidopsis thaliana*) and clover (*Medicago truncatula)* REM proteins are modified by S-acylation in their CA sequence, which contributes to their PM association but is not responsible for their nanodomain sub-compartimentalization (Konrad et al., 2014). Another post-translational modification, namely phosphorylation, influences the intracellular dynamics of REM. Thus, StREM1;3 phosphorylation in its N-terminal region modifies its lateral mobility within the PM and controls restriction of Potato Virus X cell-to-cell movement (Perraki et al., 2018). In a recent preprint, REM phosphorylation appears to be required for protein–protein interaction and is suggested to act on developmental pathways (preprint Abel et al, 2021). An important feature of REM, that governs their localization, is their propensity to oligomerize. Indeed, the assembly of REM into trimers is essential for membrane recruitment. In addition, the formation of higher-order complexes through trimer–trimer interaction was proposed to play a role in REM organization into membrane nanodomains (Bariola et al., 2004; Perraki et al., 2012; Martinez et al., 2019). On a physiological point of view, REM function was partially elucidated. These proteins were described to play fundamental roles in: (1) plant defense against virus (Raffaele et al., 2009; Fu et al., 2018; Perraki et al., 2018; Huang et al., 2019; Cheng et al., 2020) and bacteria (Albers et al., 2019), (2) symbiosis (Lefebvre et al., 2010; Liang et al., 2018), (3) hormonal signaling (Gui et al., 2016; Ke et al., 2021), (4) plant development (preprint; Abel et al., 2021).

SPFH domain-containing proteins, also called Band-7 proteins, are nanodomain-resident proteins that are evolutionary conserved and regroup different sub-families: flotillins, stomatins, prohibitins, erlins, and the plant-specific hypersensitive-induced reaction proteins (Daněk et al., 2016). Interestingly, SPFH domain-containing proteins emerged independently in different kingdoms suggesting an important function in cells (Rivera-Milla et al., 2006). How plant SPFH proteins are targeted to membranes and are recruited into nanodomains remains largely unknown. However, wild-type (WT) plants treated with the sterol-depleting agent methyl-β-cyclodextrin (mβCD) as well as the sterol biosynthesis mutant *cyclopropylsterol isomerase 1 (cpi1-1)* display altered intracellular dynamics of Flotillin 1 (Flot1; Li et al., 2012; Cao et al., 2020). In addition, flotillins possess putative sterol binding motifs named CRAC/CARC, which may participate in the recruitment of flotillins in sterol-enriched domains, although experimental evidences are still needed (Daněk et al., 2016). Lipidation might be involved in the addressing of SPFH proteins in the cell since some members of this family are modified through myristoylation and/or S-acylation (Hemsley et al., 2013; Majeran et al., 2018). Similarly to REM, SPFH proteins such as HIR are able to oligomerize (Qi et al., 2011), which could play an important role in their recruitment into membrane nanodomains or in the organization of the domains themselves. Physical interactions between SPFH proteins and REM have been reported (Arabidopsis REM1.3 and HIR1; Lv et al., 2017), as well as between different subfamilies of SPFH proteins (Arabidopsis Flotillin 2 and HIR2; Junková et al., 2018), raising the question of the importance of such protein complexes. A partial answer comes from observations showing that the recruitment of *Medicago truncatula* Symbiotic Remorin1 (SYMREM1) into nanodomains depends on FLOT4 protein that was proposed to act as a central hub during primary nanodomain assembly (Liang et al., 2018). In turn, SYMREM1 interacts with and stabilizes the activated LYSINE MOTIF KINASE 3 (LYK3) receptor into nanodomains, ensuring symbiotic root infection. Although a physical interaction between SYMREM1 and FLOT4 proteins remains to be demonstrated, these results highly suggest that interactions between members of different families of nanodomain-resident proteins is important for nanodomain organization/function. Apart from this role of some flotillins probably acting as scaffolding proteins shaping membrane nanodomains, Arabidopsis Flot1 was proposed to define a clathrin-independent endocytic pathway (Li et al., 2012; Wang et al., 2015), as detailed hereafter. Concerning the biological functions of plant SPFH proteins, flotillins were shown to play a critical role in symbiotic bacterial infection, as stated above (Haney and Long, 2010; Haney et al., 2011; Liang et al., 2018). So far, HIR proteins were reported to participate in the defense against pathogen in different plant species, although the underlying mechanisms remain misunderstood (Zhou et al., 2010; Choi et al., 2011; Qi et al., 2011; Li et al., 2019).

## Formation and organization of nanodomains

Nanodomain formation and maintenance are driven by lipid–protein interactions and by the cell wall/PM/cytoskeleton continuum. So far, it remains unclear whether a specific lipid environment is responsible for the creation of nanodomains through interactions with proteins, or at the opposite, whether some proteins, for instance nanodomain-resident proteins that bind lipids, can actively cluster lipids. Recently, nanoclusters of an anionic lipid, the phosphatidylserine, were demonstrated to control the time of residence of Rho of Plant 6 protein and consequently tune the cell response to external stimuli (Platre et al., 2019; Smokvarska et al., 2020). On the other hand, nanodomain-resident proteins such as REMs are probably able to structure their lipidic environment. Indeed, Huang et al. showed using di-4-ANEPPDHQ, a ratiometric fluorescent probe that displays different emission peak in liquid-ordered phase (nanodomain) and liquid-disordered phase (non-nanodomain), that REM1.2 overexpression induced an increase in the level of ordered lipid domains in the PM (Huang et al., 2019). In addition, solid-state nuclear magnetic resonance (NMR) analysis highlighted that the oligomerization proprieties of StREM1.3 influenced lipid order and membrane thickness (Legrand et al., 2019). By forming multimeric complexes and binding to sterols, animal SPFH domain-containing proteins were also proposed to actively participate in the formation of membrane nanodomains (Browman et al., 2007). In plants, whether the formation of SPFH protein complexes may recruit specific lipids and hence allow the formation of particular membrane nanodomains remains to be determined.

Nanodomain formation and maintenance are not limited to individual protein or lipid interactions. Indeed, structures in contact with the PM, like the cytoskeleton or the extra-cellular matrix, help or even trigger nanodomain assembly. One of the most unified models for membrane organization is coming from the “picket and fence” model (Kusumi et al., 2005). In this model, the membrane cytoskeleton, made of actin filaments, constrains the diffusion of membrane proteins by acting as fences. The membrane cytoskeleton is anchored to the membrane by fixed transmembrane proteins, the pickets, reducing the diffusion of lipids and proteins including those from the outer leaflets (Ritchie et al., 2003; Kusumi et al., 2005). Both cortical actin and microtubules act as membrane cytoskeleton, in plants. Interestingly, cytoskeleton depolymerization drugs impact the diffusion of PM localized protein such as PIN3, FLS2, Flot1, and HIR1; moreover, destabilization of actin also induces a reduction in SYMREM1 nanodomain density in cells (Lv et al., 2017; Liang et al., 2018; McKenna et al., 2019; Daněk et al., 2020). In plant, additional actors control protein diffusion. In direct contact with the PM, the cell wall constrains the lateral diffusion and organization of proteins including HIR1, Flotillin 2, PIN2, PIN3, and FLS2, as demonstrated by affecting the cell wall integrity (Feraru et al., 2011; Kleine-Vehn et al., 2011; Martinière et al., 2012; McKenna et al., 2019; Daněk et al., 2020). Therefore, the cell wall may act as fences to limit protein diffusion in the outer phase of the PM. In specific cases, cell wall may have a more direct role during signaling events. Indeed, some proteins are known to act as molecular bridges between cell wall components and actin filaments. Indeed, formins interact from one side with the cell wall, likely via their proline-rich repeat stretch in their extracellular domain, and from the other side with actin filaments via their cytoplasmic formin homology domain (Michelot et al., 2005; Martinière et al., 2011; Ma et al., 2021). A recent preprint study suggests that this transmembrane connection is necessary for symbiotic response in root hairs (preprint article; Liang et al., 2020). This exemplifies how the cell wall/PM/cytoskeleton continuum could affect cell signaling by modulating PM organization.

## Nanodomains and transport functions in plants

### Hormonal transport: PIN and ATP binding cassette transporters

Cell-to-cell transport of auxin, a phytohormone essential for plant growth and development, is mainly achieved by PM localized transporters including the polarly localized auxin efflux carriers PIN (for review, see Adamowski and Friml, 2015; Sauer and Kleine-Vehn, 2019). To tightly control the direction and the rate of auxin flow, PIN activity and intracellular trafficking/endocytosis are highly regulated. Evidences for a role of membrane nanodomains in the regulation of members of PIN family first arose from the study of sterol-biosynthesis mutants, sterol being a key component of certain membrane nanodomains. Mutation of the *STEROL METHYLTRANSFERASE1* (*SMT1*) gene disturbs sterol composition and induces aberrant auxin distribution and defective polar auxin transport in Arabidopsis resulting from the subcellular mislocalization of PIN1 and PIN3 in the root (Willemsen et al., 2003). Importantly, PIN1 polarity is altered in *smt1^orc^* mutant since, contrary to WT plants, the protein is no more exclusively localized in the basal PM of central vascular cells but is also detected in lateral PMs. The importance of sterol composition in PIN3 localization is in accordance with PIN3 protein being present in Arabidopsis DRM fractions (Demir et al., 2013). Similarly to *smt1^orc^*, in the Arabidopsis *cyclopropylsterol isomerase 1 (cpi1-1)* mutant, which displays a modified sterol profile, polar PIN2 is redistributed to ectopic PM domains i.e. lateral PM in cortical cells and basal PM in epidermal cells (Men et al., 2008). Consistently, *cpi1-1* displays disturbed auxin distribution and default in root gravitropism. Important insight in the regulation of PIN2 by sterols came from the observation that PIN2 failed to be properly endocytosed in *cpi1-1* background. The importance of sterols in PIN2 endocytosis was confirmed by showing that the sterol-binding agent filipin, which induces sterol desorption, reduces PIN2 internalization from the PM (Men et al., 2008). Since endocytosis is one of the mechanisms involved in the maintenance of PIN polarity (Kitakura et al., 2011; Kleine-Vehn et al., 2011; Glanc et al., 2018), the defect of PIN2 endocytosis in *cpi1-1* mutant is likely responsible, at least in part, for its aberrant subcellular distribution. Semi-quantitative confocal and super-resolution microscopy revealed that PIN1 and PIN2 accumulate in clusters of 100–200 nm in diameter at the PM and that the organization of these nanodomains is disturbed by filipin, showing a role of sterols in their establishment/maintenance (Kleine-Vehn et al., 2011). Filipin treatment results in an increase in membrane lateral mobility of PIN2 and ultimately alters PIN2 polarity, suggesting that the nanodomain organization of PIN2 limits its lateral diffusion and thus is involved in the maintenance of its polarity (Kleine-Vehn et al., 2011). Intriguingly, the lateral diffusion of PIN2 in the PM is not affected in *cpi1-1* (Men et al., 2008) highlighting differences observed between sterol-biosynthesis mutants and the use of sterol depleting agents and cautions that should be taken while studying the role of sterols and membrane nanodomains in plants. Another level of complexity in our understanding of the role of sterols and possibly membrane nanodomains in the regulation of PIN intracellular dynamics came from a study showing that sterols play a key role in the auxin-induced inhibition of PIN2 endocytosis in roots (Pan et al., 2009). Indeed, PIN2 is no more stabilized at the PM in response to auxin in the sterol biosynthetic mutants cotyledon vascular pattern1 (*cvp1-3*) and fackel-J79 (*fk-J79)* or when WT plants are treated with the chemical compound fenpropimorph that disrupts sterol biosynthesis. Interestingly, sterol levels were shown to be reduced in auxin signaling mutants in which auxin-induced inhibition of PIN2 endocytosis is abrogated, supporting a link between sterols and auxin signaling in the regulation of PIN2 endocytosis (Pan et al., 2009). The negative effect of sterols on PIN2 endocytosis described by Pan and co-workers appears relatively contradictory with previous work showing that sterols are required for constitutive internalization of PIN2 from the PM (Men et al., 2008). One possible explanation is that sterols may play different roles between constitutive and auxin-regulated PIN endocytic mechanisms. Apart from sterols, a recent study suggests that other lipids such as phosphoinositides regulate PIN2 nanoclustering which also depends on microtubules, cell-wall components and connections between the PM and the cell wall (Li et al., 2021).

Interestingly, auxin was recently shown to induce sterol-dependent nanoclustering of proteins in the PM during the morphogenesis of Arabidopsis cotyledon pavement cells (Pan et al., 2020). Ordered membrane domains are preferentially localized to indenting regions of pavement cells as demonstrated by using di-4-ANEPPDHQ. In addition, diverse sterol biosynthetic mutants or WT plants treated with mβCD display defects in pavement cell morphogenesis, leading the authors to propose that sterols and nanodomains are important for establishing the indenting regions of pavement cells. Auxin was shown to promote the formation of the puzzle-piece pavement cell shape (Xu et al., 2010). Interestingly, di-4-ANEPPDHQ staining revealed that the auxin biosynthesis mutant *weak ethylene insensitive 8/Tryptophan aminotransferase related 2 wei8-1/tar2-1* exhibits reduced lipid ordering in pavement cells, which is reverted by exogenous auxin (Pan et al., 2020). A role for auxin in the regulation of membrane nanodomains was reinforced by TIRF microscopy analyses showing that auxin treatment increased the size of Flot1-containing nanodomains, this pathway being dependent on the Transmembrane Receptor Kinase 1 and 4 (TMK1 and TMK4; Pan et al., 2020). Interestingly, the salicylic acid (SA) hormone also modulates nanodomain organization by increasing the liquid-ordered phase of the PM (Huang et al., 2019). In addition, SA was demonstrated to impair PIN2 lateral diffusion and internalization through CME, inducing the formation of PIN2 hyper-clusters, which results in a loss of the root gravitropic response (Ke et al., 2021). This mechanism involves the nanodomain-resident protein REM1.2 where clustering is regulated by SA (Huang et al., 2019; Ke et al., 2021).

How PIN proteins are recruited in membrane nanodomains remains largely unknown. However, PIN1 nanodomain localization was proposed to be achieved through an interaction with an ATP binding cassette (ABC) transporter from the B subgroup named ABCB19 (Titapiwatanakun et al., 2009). ABCB19 transports auxin and physically interact with PIN1 to coordinately regulate auxin efflux (Blakeslee et al., 2007; Mravec et al., 2008). In addition, ABCB19 was detected in DRM, suggesting that it is present in membrane nanodomains (Titapiwatanakun et al., 2009). In WT plants, PIN1 protein is found in both DRM and Triton X-100-sensitive fractions, whereas in *abcb19* mutant, PIN1 is absent of DRM, suggesting that ABCB19 recruits PIN1 in this membrane fraction (Titapiwatanakun et al., 2009). Now, microscopy approaches are needed to better understand how ABCB19 handles the nanodomain organization of PIN1 in membranes. On a functional point of view, the recruitment of PIN1 and ABCB19 in certain membrane nanodomains may regulate their activity since sterol supply was shown to slightly increase auxin transport induced by ABCB19 or the combination of ABCB19/PIN1 expressed in a heterologous system (Titapiwatanakun et al., 2009). Besides ABCB19 protein, other ABC transporters were identified in DRM prepared from Arabidopsis tissues such as ABCB1, ABCB4, and ABCB21 that transport auxin as well as ABCG36/PEN3/PDR8 that acts as an efflux pump of cadmium but that also possibly facilitates the transport of compounds involved in the defense against pathogens (Borner et al., 2005; Terasaka et al., 2005; Geisler and Murphy, 2006; Stein et al., 2006; Kim et al., 2007; Kamimoto et al., 2012; Demir et al., 2013). So far, the localization of these ABC transporters in plant membrane nanodomains remains to be established by microscopy as well as the biological outcome of such a putative clusterization.

### Water and ions

#### Aquaporins and water transport

Aquaporins are channel proteins ubiquitously present in cell membranes. They are historically known for water transport, but can also facilitate the diffusion of small uncharged molecules like hydrogen peroxide (H_2_O_2_), ammonia, glycerol, or carbon dioxide (Maurel et al., 2015). In Arabidopsis, four subfamilies of aquaporins are found, including the PIPs that are divided into two subgroups, PIP1 and PIP2. Many PIP isoforms have been identified in DRM fractions from different plant species (Table 1), and some fluorescently tagged PIP were also revealed to display a dotted localization in the PM using TIRF microscopy (Minami et al., 2009; Li et al., 2011; Demir et al., 2013). The subcellular localization of PIP2;1, the most abundant root aquaporin in Arabidposis, was demonstrated to be highly dynamic in response to variations of the cell environment. In control conditions, PIP2;1 cycles between the PM and endosomes, whereas it is internalized from the PM following salt or osmotic stresses (Luu et al., 2012; Luu and Maurel, 2013), which is associated with a decrease in the root hydraulic conductivity (Lpr; Boursiac et al., 2005, 2008). PIP2;1 depletion from the PM is in part achieved through CME since (1) PIP2.1 partially co-localizes with CLC2 protein and (2) overexpression of a dominant negative Clathrin Heavy Chain or treatment with auxin 1-naphthaleneacetic acid, which reduces CME in root tips, inhibit PIP2;1 internalization (Li et al., 2011; Luu et al., 2012; Martinière et al., 2019). However, PIP2;1 endocytosis also occurs independently of the clathrin machinery through a nanodomain-mediated pathway. Indeed, upon a salt stress (100-mM NaCl), the co-localization between PIP2;1 and the nanodomain-resident protein Flot1 increases (Li et al., 2011). In addition, depletion of sterol with mβCD, treatments with the sterol synthesis inhibitor fenpropimorph, or with DL-threo-1-phenyl-2-palmitoylamino-3-morpholino-1-propanol, a sphingolipid biosynthesis inhibitor, reduce PIP2;1 internalization, suggesting a role of these lipids in PIP2;1 endocytosis (Li et al., 2011). Altogether, these results show that PIP2;1 depletion from the PM is tightly associated with PIP2;1 organization into nanodomains. Super-resolution approaches gave important information regarding the organization of PIP2;1 individual molecules in nanodomains. Thus, single molecule localization and voronoi image segmentation showed that PIP2;1 is present in domains of nanometric size in the PM (Martinière et al., 2019). In addition, molecule tracking by sptPALM revealed that an hyperosmotic treatment (300-mM sorbitol) induced PIP2;1 diffusion but also enhanced its local density, concomitantly to an increase in PIP2;1 internalization in root cells (Martinière et al., 2019). These results suggest that PIP2;1 enrichment in nanodomains in response to an osmotic stress may reflect a recruitment in foci at the PM before endocytosis. Interestingly, this response is specific to PIP2;1, since other PM localized proteins, like the proton pump H^+^-ATPase2 (AHA2), do not show an increase in endocytosis nor in their local protein density under similar osmotic condition. PIP2;1 dynamics and dwell time were also analyzed in leaves during stomata immune response. Aquaporin nanodomain diffusion in response to the bacterial elicitor flg22 appears to be specific to stomata cell and absent in subsidiary cells (Cui et al., 2021).

**Table 1:** Various aquaporin isoforms identified by mass spectrometry in DRM fractions from different plant species.

Accessions	Proteins	Species	Tissues	References
*AT3G61430*	AtPIP1;1	*Arabidopsis thaliana*	Callus from roots/whole plant/leaves	Borner et al., 2005; Minami et al., 2009; Demir et al., 2013
*AT2G45960*	AtPIP1;2	*Arabidopsis thaliana*	Callus from roots/whole plant/leaves	Borner et al., 2005; Minami et al., 2009; Demir et al., 2013
*AT1G01620*	AtPIP1;3	*Arabidopsis thaliana*	Leaves	Demir et al., 2013
*AT4G00430*	AtPIP1;4	*Arabidopsis thaliana*	Whole plant/leaves/suspension cell cultures	Minami et al., 2009; Demir et al., 2013; Szymanski et al., 2015
*AT4G23400*	AtPIP1;5	*Arabidopsis thaliana*	Whole plant/leaves	Minami et al., 2009; Demir et al., 2013
*AT3G53420*	AtPIP2;1	*Arabidopsis thaliana*	Whole plant/leaves	Minami et al., 2009; Demir et al., 2013
*AT2G37170*	AtPIP2;2	*Arabidopsis thaliana*	Whole plant/leaves	Minami et al., 2009; Demir et al., 2013
*AT2G37180*	AtPIP2;3	*Arabidopsis thaliana*	Whole plant/leaves	Minami et al., 2009; Demir et al., 2013
*AT3G54820*	AtPIP2;5	*Arabidopsis thaliana*	Whole plant	Minami et al., 2009
*AT2G39010*	AtPIP2;6	*Arabidopsis thaliana*	Whole plant/leaves	Minami et al., 2009; Demir et al., 2013
*AT4G35100*	AtPIP2;7	*Arabidopsis thaliana*	Callus from roots/whole plant/suspension cell cultures/leaves	Borner et al., 2005; Minami et al., 2009; Keinath et al., 2010; Demir et al., 2013; Yoshida et al., 2013
*AT2G16850*	AtPIP2;8	*Arabidopsis thaliana*	Suspension cell cultures	Yoshida et al., 2013
*AT3G26520*	AtTIP1;2	*Arabidopsis thaliana*	Leaves	Demir et al., 2013
*AT3G16240*	AtTIP2;1	*Arabidopsis thaliana*	Leaves	Demir et al., 2013
*AAK66766.1*	MtPIP1;1	*Medicago truncatula*	Roots	Lefebvre et al., 2007
*AAL32127*	MtPIP2;1	*Medicago truncatula*	Roots	Lefebvre et al., 2007
*Q40595*	Aquaporin	*Nicotiana tabacum*	BY2 cells	Morel et al., 2006
*Q8W506*	NtPIP2;1	*Nicotiana tabacum*	BY2 cells	Morel et al., 2006
*O24662*	NtAQP1	*Nicotiana tabacum*	BY2 cells	Morel et al., 2006
*AK072519*	OsPIP2;1	*Oryza sativa*	Suspension cell cultures	Ishikawa et al., 2015
*AK103807*	OsPIP1;1	*Oryza sativa*	Suspension cell cultures	Ishikawa et al., 2015

PIP and other aquaporin members are known to facilitate the diffusion across membranes of H_2_O_2_ that acts as a signaling molecule in plant cells (Dynowski et al., 2008; Bienert et al., 2012). For instance, intracellular H_2_O_2_ accumulation in response to the phytohormone ABA or the bacterial elicitor flg22 induces stomata closure (Rodrigues et al., 2017). ABA and flg22 activate PM-localized NADPH oxidases, leading to the accumulation of superoxide in the apoplasm, which turns to H_2_O_2_ through dismutation. Similarly to PIP2;1, the NADPH oxidase RbohD was shown to be present in DRM fractions and to cluster in nanodomains, as revealed by microscopy approaches (Hao et al., 2014). Interestingly, the entrance of H_2_O_2_ into guard cells is mediated by PIP2;1 in a phosphorylation-dependent manner (Rodrigues et al., 2017). Indeed, phosphomimic or phosphodead mutations of Ser121 residue in PIP2;1 enhance or reduce cell permeability to H_2_O_2_, respectively. The two kinases Open Stomata 1 (OST1)/Snf1-related protein kinase 2.6 (SNRK2.6) and BRI 1-associated receptor kinase 1 (BAK1) were shown to phosphorylate PIP2;1 on Ser121 residue in vitro. Interestingly, the same kinases also target NADPH oxidases (Mustilli et al., 2002; Li et al., 2014), suggesting that the production and the transport of reactive oxygen species (ROS) might be co-regulated. The eventual role of membrane nanodomains acting as a scaffold to generate a putative Rboh/PIP complex remains an open question. ROS are known to modify PIP2;1 nanodomain organization and dynamics. Indeed, TIRF microscopy revealed that exogenous application of H_2_O_2_ induces both PIP2;1 nanodomain diffusion and endocytosis, linking again PIP2;1 nanodomain dynamics and protein cycling (Wudick et al., 2015). However, when plant cells are stimulated to accumulate intracellular H_2_O_2_ (e.g. under an osmotic stress), H_2_O_2_ appears to act as a negative regulator of PIP2.1 diffusion (Martinière et al., 2019). This result is counterintuitive and might reflect that where ROS are produced or accumulate has a great impact on the way it acts on protein diffusion. In addition to localization aspects, endogenous H_2_O_2_ production and exogenous application differ in their concentrations, which could have different impacts on membranes. Interestingly, ROS were shown to modify membrane biophysics. Indeed, after cell stimulation by cryptogein, an oomycete pathogen elicitor, ROS produced by tobacco (*Nicotiana tabacum*) RbohD increase lipid ordering in the membrane (Noirot et al., 2014; Sandor et al., 2016).

In mammalian cells, lipid environment can directly modify aquaporin activity as demonstrated for AQP4 and AQP0 (Tong et al., 2013, 2016). Especially the ratio between phospholipids and proteins was shown to modify the water permeability induced by these aquaporins. This supposes that protein lipid hydrophobicity matching acts on aquaporin activity (Kim et al., 2020). In addition, AQP4 and AQP2 activities are tightly linked to cholesterol concentration in the membrane (Tong et al., 2012, 2013). Cholesterol was proposed to have an impact on membrane thickness that acts on both aquaporin pore length and protein organization near the cytoplasmic vestibule of the pore (Tong et al., 2016). In plants, the role of lipids on aquaporin activity remains largely understudied. However, using liposomes, sterols were shown to have a negative effect on water and CO_2_ transport mediated by NtPIP2;1 and NtAQP1, respectively (Kaldenhoff et al., 2014). These observations make plausible that the recruitment of aquaporins into nanodomains that display a specific lipid composition, influences their activity.

#### Ion transport and nanodomains

The potassium channel KAT1 was one of the first proteins observed in dotted structures in the PM, corresponding to nanodomains (Sutter et al., 2006; Homann et al., 2007; Reuff et al., 2010; Jarsch et al., 2014). Although some shaker channels are known to be regulated by lipid environment in animal cells, the role of KAT1 organization into nanodomains on potassium uptake remains elusive (Romanenko et al., 2002; Martens et al., 2004). At the opposite, the function of nanodomains in nitrogen acquisition is more understood. Ammonium is one of the sources of nitrogen in plants, but it becomes toxic at high concentration. Therefore, its uptake and metabolism have to be tightly controlled in the cell. AMTs are responsible for about 90% of the NH4^+^ uptake in roots (Yuan et al., 2007). Variable-angle TIRF analysis revealed that AMT1;3 forms discrete foci of various intensity and resident time in the PM of root cells (Wang et al., 2013). Interestingly, plants exposed to high ammonium levels show a decrease in AMT1;3-GFP PM labeling, a reduced density of nanodomains, but a significant increase in their size, suggesting an enhanced AMT1;3 internalization. The authors showed that in a mutant for an isoform of the glutamine synthase1 (GLN1;2), a key enzyme in N metabolism and recycling, the size of AMT1;3-GFP nanodomains was enhanced compared to WT plants in low NH4 conditions and that the overall fluorescence intensity of AMT1;3-GFP in the PM was reduced. Since *gln1.2* accumulates higher intracellular NH4^+^ levels, this result suggests that AMT1;3 internalization is regulated by the internal ammonium concentration (Wang et al., 2013). The depletion of AMT1;3-GFP from the PM and its degradation under high ammonium condition is reduced in *Clathrin heavy Chain 2* (*CHC2*) mutant, showing a role of CME in this process. However, a clathrin-independent pathway is also involved in ammonium-mediated internalization of AMT1;3 since both Flot1miRNA lines and WT plants treated with mβCD showed more AMT1;3-GFP signal at the PM than controls under high ammonium condition. This result is in accordance with the partial co-localization observed between Flot1-mCherry and AMT1.3-GFP in nanodomains (Wang et al., 2013). Similarly to AMT1;3, the nitrate transporter NRT1.1 fused to fluorescent proteins forms sub diffraction spots at the PM, as determined by variable-angle TIRF microscopy (Zhang et al., 2019). NRT1.1 nitrate transport activity and signaling are governed by the phosphorylation of T101 residue. Interestingly, NRT1.1^T101D^ phosphomimetic version displays a fast nanodomain diffusion in the PM, whereas the NRT1.1^T101A^ is less diffusible. Similarly to AMT1;3, clathrin-mediated and -independent endocytosis seem to be both needed for NRT1.1 internalization and are regulated by NRT1.1 phosphorylation status (Zhang et al., 2019). The similarity between the internalization of AMT1;3 and NRT1.1 may suggest a co-regulation by cell nitrogen status. Zhang et al. (2019) also showed by TIRF microscopy that the fast diffusible NRT1.1^T101D^ variant interacts in planta with the nanodomain-resident protein AtREM1.3. This result is intriguing since REM-positive nanodomains should contain ordered lipids inducing local high viscosity of the membrane and, consequently, the proteins present in these domains are supposed to have a slow diffusion. Similar apparent discrepancy between protein diffusion and localization in DRM or co-localization with nanodomain-resident proteins was previously already reported by the same group (Xue et al., 2018). This illustrates probably two distinct phenomena, e.g. diffusion of a nanodomain within the PM recorded by TIRF and diffusion of individual proteins within a nanodomain only accessible by SMLM.

In addition to being involved in protein internalization from the PM, membrane nanodomains probably constitute a “basement” for protein complex assembly required for the regulation of ion uptake. Indeed, the anion channel Slow Anion Channel 1 Homolog 3 (SLAH3) interacts with its regulatory kinase Calcium-Dependent Protein Kinase 21 (CPK21) in PM nanodomains, as revealed by bimolecular fluorescence complementation, and the co-expression of SLAH3 and CPK21 redirects both proteins from detergent-sensitive membranes (DSM) to DRM (Demir et al., 2013). Importantly, ABA treatment increases the physical interaction between SLAH3 and CPK21, as demonstrated by Förster resonance energy transfer technique. Interestingly, upon co-expression with the Protein Phosphatase 2C/abscisic-acid insensitive 1 (PP2C/ABI1), the SLAH3/CPK21 complex dissociates that corresponds with a loss of SLAH3 activity. Altogether, these data illustrate the role of nanodomains in the assembly or the dissociation of protein complexes and their impact on protein function in response to cell stimuli. The incidence of environmental cues on the recruitment of proteins in nanodomains/DRM is illustrated by a recent study on Calcineurin B-like (CBL)-type Ca^2+^ sensors that regulate CBL-interacting protein kinases (Chu et al., 2021). Genetic depletion of the five PM-localized CBL was shown to alter root growth and nitrate uptake. In addition, phosphoproteomic study and membrane fractionations demonstrated that CBL control both the phosphorylation of numerous PM proteins e.g. NRT2.1, PIPs, AHAs, and their distribution in DRM/DSM fractions in response to nitrate status. These results suggest a functional link between calcium signaling, protein phosphorylation, and repartition of proteins in DRM.

### Nanodomains and transport in plants: what is next?

In plants, the interconnections between membrane nanodomains and the transport of molecules is probably not restricted to the few cases described so far (see above), as attested by the diversity of transporters and channels identified by proteomic analysis in DRM and hence potentially present in nanodomains. Of course, for several reasons including the lipid composition of the nanodomain itself, the stability of the association of a given protein with nanodomains or experimental settings such as the sensitivity of protein detection, DRM data do not provide an exhaustive list of transport proteins that cluster into nanodomains. This is exemplified by the fact that only a small fraction of Arabidopsis membrane proteome was demonstrated to be present in DRM, so far. Nevertheless, proteomic analysis of DRM still gives an interesting starting point for further characterizations. Therefore, we provide here a nonexhaustive list of Arabidopsis transport proteins identified in DRM and classified according to the transported molecules (Table 2). Sugar transporters are well represented in Arabidopsis DRM fractions. The sucrose transporter StSUT1 is known for a long time to be associated with DRM fraction and StSUT1 was proposed to cluster in PM nanodomains (Krügel et al., 2008, 2012). However, so far, the significance of this localization remains to be determined. Interestingly, an important proportion of proteins listed in Table 2 transport molecules such as metals, phosphate or calcium, which suggests a still unsuspected role of membrane nanodomains in plant mineral nutrition. As stated earlier in this review, DRM biochemical approaches can provide interesting trails to follow, but further investigations are needed to confirm by microscopy approaches the presence of these proteins in membrane nanodomains and to analyze the impact of such a localization on their functioning as well as the physiological outcome.

**Table 2:** Nonexhaustive list of transport-associated proteins identified by mass spectrometry in Arabidopsis DRM fractions prepared from different tissues. Transported molecules are indicated according to gene ontogeny annotations from The Arabidopsis Information resource (TAIR, https://www.arabidopsis.org). In the category “Metals”, note that the transported metal is indicated in parenthesis in the accession column.

Transported molecules	Protein/Protein family	Accessions	Tissues	References
*Amino acids*	Cationic amino acid transporter (CAT)	AT4G21120 (CAT1)	Suspension cell cultures	Keinath et al., 2010
	Lysine histidine transporter (LHT)	AT5G40780 (LTH1)	Suspension cell cultures	Keinath et al., 2010
*Ammonium*	Ammonium transporter (AMT)	AT4G13510 (AMT1;1) ; AT2G38290 (AMT2)	Suspension cell cultures/leaves	Keinath et al., 2010; Demir et al., 2013
*Anions*	Voltage-dependent anion channel (VDAC)	AT5G67500 (VDAC2) ; AT5G15090 (VDAC3)	Callus from roots/ Suspension cell cultures	Borner et al., 2005; Keinath et al., 2010
	Mechanosensitive channel of small conductance-like (MSL)	AT5G12080 (MSL10)	Suspension cell cultures	Yoshida et al., 2013
	Chloride channel (CLC)	AT5G33280 (CLCG)	Suspension cell cultures	Yoshida et al., 2013
*Ascorbate*	Nucleobase-ascorbate transporter (NAT)	AT2G27810 (NAT12) ; AT1G60030 (NAT7)	Suspension cell cultures	Keinath et al., 2010
*Calcium*	Autoinhibited Ca^2+^-ATPase (ACA)	AT1G27770 (ACA1) ; AT2G41560 (ACA4) ; AT5G57110 (ACA8) ; AT4G29900 (ACA10)	Suspension cell cultures/leaves	Keinath et al., 2010; Demir et al., 2013; Yoshida et al., 2013
	Sodium/calcium exchanger	AT1G53210	Suspension cell cultures	Szymanski et al., 2015
	Two-pore channel 1	AT4G03560	Suspension cell cultures	Yoshida et al., 2013
	Auto-regulated calcium ATPase (ACA)	AT2G22950 (ACA7)	Suspension cell cultures	Yoshida et al., 2013
*Cation*	Early-responsive to dehydration (ERD)	AT1G30360 (ERD4)	Leaves	Demir et al., 2013
*Glucosinolate*	Glucosinolate transporter (GTR)	AT3G47960 (GTR1)	Suspension cell cultures	Keinath et al., 2010
*Lipids*	Aminophospholipid ATPase (ALA)	AT5G44240 (ALA2)	Suspension cell cultures	Yoshida et al., 2013
	Glycosylphosphatidylinositol-anchored lipid protein transfer (LTPG)	AT1G27950 (LTPG1)	Callus from roots	Borner et al., 2005
*Metals*	Pleitropic drug resistance (PDR)	AT1G59870 (PDR8, cadmium)	Leaves	Demir et al., 2013
	Copper transporter (COPT)	AT5G20650 (COPT5, copper)	Suspension cell cultures	Yoshida et al., 2013
	Magnesium transporter (MGT)	AT1G16010 (MGT2, magnesium)	Suspension cell cultures	Yoshida et al., 2013
	Natural resistance-associated macrophage protein (NRAMP)	AT2G23150 (NRAMP3, manganese/iron)	Suspension cell cultures	Yoshida et al., 2013
	Oligopeptide transporter (OPT)	AT4G16370 (OPT3, iron)	Leaves	Demir et al., 2013
	Yellow stripe like (YSL)	AT1G65730 (YSL7, metals/bacterial factors)	Suspension cell cultures	Keinath et al., 2010
	Metal tolerance protein (MTP)	AT2G46800 (MTP1)	Suspension cell cultures	Yoshida et al., 2013
*Nitrate*	Nitrate transporter (NRT)	AT5G50200 (NTR3.1); AT1G52190 (NTR1.11)	Suspension cell cultures/leaves	Keinath et al., 2010; Demir et al., 2013
*Nucleosides*	Equilibrative nucleoside transporter (ENT)	AT4G05120 (ENT3)	Suspension cell cultures	Keinath et al., 2010
*Peptides*	Peptide transporter (PTR)	AT3G54140 (PTR1); AT2G02040 (PTR2)	Suspension cell cultures	Keinath et al., 2010; Yoshida et al., 2013
	Oligopeptide transporter (OPT)	AT5G64410 (OPT4)	Suspension cell cultures	Keinath et al., 20100
*Phosphate*	Phosphate transporter (PHT)	AT5G43350 (PHT1;1); AT2G38940 (PHT1;4); AT3G54700 ((PHT1;7)	Suspension cell cultures	Kierszniowska et al., 2009; Keinath et al., 2010
	Non-intrinsic ABC protein (NAP)	AT1G67940 (NAP3)	Suspension cell cultures	Yoshida et al., 2013
*Sugar*	Sugar transporter protein (STP)	AT1G11260 (STP1); AT3G19930 (STP4); AT5G26340 (STP13)	Whole plant/suspension cell cultures/leaves	Minami et al., 2009; Keinath et al., 2010; Demir et al., 2013
*Sugar (continued)*	Sucrose transporter (SUT)	AT1G22710 (SUT1)	Leaves	Demir et al., 2013
	Sucrose-proton symporter (SUC)	AT1G71880 (SUC1)	Leaves/suspension cell cultures	Demir et al., 2013; Yoshida et al., 2013
	Polyol/monosaccharide transporter (PLT)	AT3G18830 (PLT5)	Leaves	Demir et al., 2013
	Tonoplast monosaccharide transporter (TMT)	AT4G35300 (TMT2)	Suspension cell cultures	Yoshida et al., 2013
	Vacuolar glucose transporter (VGT)	AT3G03090 (VGT1)	Suspension cell cultures	Yoshida et al., 2013

## Conclusion and prospects

A number of proteins transporting molecules such as auxin, water, nitrate, and ammonium across the PM were demonstrated both to be associated with DRM and to form punctuated structures in the PM when fused to fluorescent proteins. Whereas a role of nanodomains in the endocytosis of different cargo proteins in response to cell stimulus is clearly established, a putative function of nanodomains acting as scaffolding units in the PM remains largely unexplored. In the case of cell signaling, reports suggest that nanodomains can serve to separate different membrane proteins to ensure signal specificity. This is exemplified by the immune receptor FLS2 and the brassinosteroid receptor BRI1 that are involved in different biological processes while employing common downstream signaling components and that segregate into distinct PM nanodomains labeled with different REM proteins (Bücherl et al., 2017). Whether BAK1, the downstream co-receptor of FLS2 and BRI1, is recruited in one or the other nanodomain population to trigger the appropriate response remains an open question. Interestingly, some membrane proteins seem to control the segregation of other proteins in nanodomains. Thus, FERONIA, a membrane receptor belonging to the *Catharanthus roseus* receptor like kinase family, was demonstrated to act as a scaffold for FLS2 allowing the maintenance of the pathogen triggered immunity (Stegmann et al., 2017). In addition, a recent preprint suggests that FLS2 PM nanoscale dynamics is dependent on FERONIA, but neither FERONIA kinase activity nor the deletion of a part of its extracellular domain are needed to regulate immune signaling (Gronnier et al., 2020). This illustrates how a single protein can act on membrane nano-organization and might nucleate receptor complexes to orchestrate signaling responses. Concerning transport functions, similar processes based on protein exclusion/recruitment were suggested for the regulation of SLAH3 by CPK21 (Demir et al., 2013). We can wonder how such mechanisms can be extrapolated to other channels and transporters.

**Figure 2:**
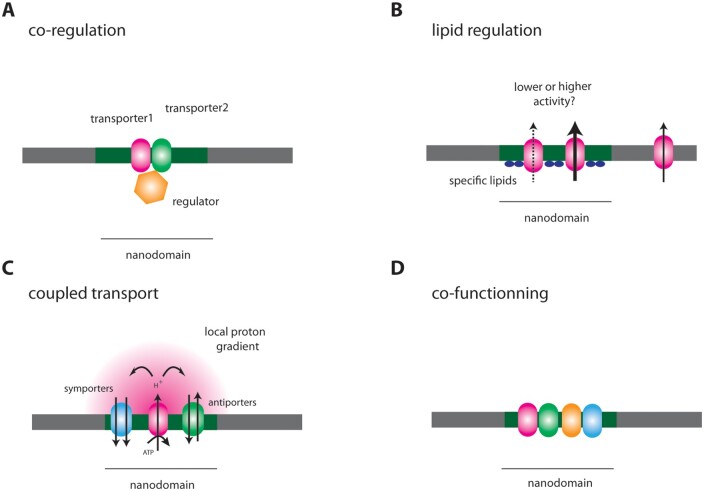
Putative roles of nanodomains in the regulation of transport functions in plants. Note that most of those roles have not been formally proven and still need to be explored. A, Nanodomain-grouped proteins (green and red transporters) are controlled by a unique regulator to coordinate a given function. B, Nanodomain lipid composition regulates transport activity (either an increase or a decrease). Consequently, this activity would vary if the transporter is in or out of the nanodomain. C, Because of the high diffusion constant of small molecules and ions, a steep gradient of concentration is created near transporters. In the drawing, we take the example of the P-type ATPase. This local gradient of protons can be used by antiporters or symporters. D, A set of functionally interconnected proteins can be grouped in a given nanodomain to optimize a cellular process. This could ensure a coordinated function like in the case of the acidification–reduction–transport strategy of iron uptake in Arabidopsis

Contrary to the segregation mechanism described above for FLS2 and BRI1, nanodomains might allow protein co-regulation, with a single regulator targeting different proteins within the same nanodomain (see Outstanding Questions, Figure 2A). As stated earlier in this review, kinases involved in the NADPH oxidase RbohD activation and PIP2;1 regulation are the same (Mustilli et al., 2002; Li et al., 2014; Rodrigues et al., 2017). Thus, it is tempting to speculate that certain isoforms of aquaporins and NADPH oxidases localize in the same PM nanodomains for co-regulation, to ensure coordination between ROS production and H_2_O_2_ transport into the cell.

Nanodomains display specific lipid compositions, which locally affect membrane biophysical properties. For instance, in model membranes and by making simulations, enrichment in saturated fatty acids or cholesterol induces a more viscous environment and a thicker membrane, which in turn impacts lateral sorting of proteins (Kaiser et al., 2011; Ernst et al., 2016). Because a perfect hydrophobic match between amino acids of protein transmembrane domains and the lipid bilayer has to be maintained, changes in membrane thickness act on protein 3D structure and, consequently, may affect channel and transporter activities. Although only speculations can be done at this stage, we can imagine that the nanodomain lipid composition may directly affect protein activity. Consequently, whether a given protein is localized in or out specific nanodomains may influence its activity (Figure 2B). This aspect of the role of nanodomains in plant transport function is mostly unexplored. Especially, the existence of nanodomains with different lipid compositions remains to be validated in plants. However, such a regulation would constitute an outstanding way to couple protein membrane dynamics, signaling, and transport activities in the PM. Interestingly, a link between lipids and calcium transport has been suggested in a work on salt sensing in plant. Glycosyl inositol phosphoryl ceramides (GIPC), lipids present in the external leaflet of the PM and enriched in DRM, were shown to bind apoplastic Na^+^ (Cacas et al., 2013; Gronnier et al., 2017; Jiang et al., 2019). This GIPC/Na^+^ complex was suspected to be involved in the gating of putative Ca^2+^ channels that are activated during salt stress to initiate an appropriate cellular response.

The ways nanodomains act on channels and transporters in plant are probably multiple. Hereafter, we will speculate on two putative roles. First, ion transport is coupled to the electrochemical gradient across the membrane, often driven by the proton motive force. Because of the speed of diffusion, a steep gradient of proton concentration is produce around proton pumps. The presence of transporters in their close vicinity, such as in the same nanodomain, may favor molecule transport (Figure 2C). Second, the concept of protein proximity among specific nanodomains allowing the optimization of molecule transport could be pushed further. Indeed, the assembly of protein complexes may allow a local cooperation between proteins with interconnected functions (Figure 2D). Recently, the iron transporter named Iron Regulated Transporter1, the Ferric Reduction Oxydase2 reductase, and the proton pump AHA2, that work in concert in the acidification–reduction–transport strategy of iron uptake in Arabidopsis, were shown to physically interact in the PM (Barranco et al., 2020). This iron-acquisition complex was proposed to optimize root iron uptake. Whether such a complex is formed in specific PM nanodomains remains to be determined.

The way protein organization into PM nanodomains controls transport functions just started to be explored in plants, but this topic will undeniably provide exciting insights in our understanding of the regulation of water and ion uptake and plant response to the environment.

**Outstanding questions**

- How are plant nanodomains formed and maintained?
- How are plant nanodomains modified by cell nutrition signals?
- How can nanodomain lipid composition regulate transporter activity?
- Could nanodomains act as functional units in membranes by gathering proteins with interdependent functions?

## Notes

A.M. and E.Z. wrote the manuscript.

The author responsible for distribution of materials integral to the findings presented in this article in accordance with the policy described in the Instructions for Authors (https://academic.oup.com/plphys/pages/general-instructions) is: Alexandre Martinière (alexandre.martiniere@cnrs.fr).
